# Association Between the Presence of Pulmonary Hypertension Before Cardiovascular Surgery and the Nephroprotective Effect of Carperitide: A Retrospective Cohort Study

**DOI:** 10.7759/cureus.22891

**Published:** 2022-03-06

**Authors:** Shin Suzuki, Yoshitaka Aoki, Hiroki Anezaki, Chiharu Wakuda, Shinji Oshima, Hisako Nishimoto, Atsushi Kobayashi, Hiromi Kato, Matsuyuki Doi, Yoshiki Nakajima

**Affiliations:** 1 Department of Anesthesiology and Intensive Care Medicine, Hamamatsu University School of Medicine, Hamamatsu, JPN

**Keywords:** creatinine, urine, renal replacement therapy, pulmonary hypertension, acute kidney injury, carperitide, atrial natriuretic peptide

## Abstract

Introduction

We hypothesized that the nephroprotective and diuretic effects of carperitide are effective in patients with pulmonary hypertension. We examined the presence of preoperative pulmonary hypertension and the effects of carperitide.

Methods

In this retrospective cohort study, we included patients aged 20 years or older who received carperitide during cardiovascular surgery and were admitted to the postoperative intensive care unit. We used hospital data from March 2019 to September 2021. The outcomes were the incidence of acute kidney injury, the number of patients using renal replacement therapy in the intensive care unit, urine volume in the first 24 hours after surgery, and the difference in serum creatinine concentrations between before and after surgery. After adjusting for confounding factors by multivariate analysis, we compared the difference in outcomes with and without preoperative pulmonary hypertension (systolic pulmonary artery pressure ≥36 mmHg).

Results

The study included 244 patients, with 72 (29.5%) in the pulmonary hypertension group and 172 (70.5%) in the control group. Acute kidney injury occurred in eight (11.1%) patients in the pulmonary hypertension group and in 18 (10.5%) patients in the control group, with no significant difference by logistic regression analysis (odds ratio 1.40, 95% confidence interval 0.54-3.62, p=0.49). Additionally, the use of renal replacement therapy, urine volume at 24 hours postoperatively, and the difference in serum creatinine concentrations were not different between the two groups.

Conclusions

Our results suggest that the effect of carperitide during cardiovascular surgery is not affected by the presence or absence of pulmonary hypertension.

## Introduction

Acute kidney injury (AKI) remains a common complication after cardiovascular surgery [1,2]. The mechanisms of AKI appear to be multifactorial, including genetic predisposition, nephrotoxins, cardiopulmonary bypass-induced hemolysis, ischemic-reperfusion injury, complexity of cardiac surgery, oxidative stress, and inflammation [2,3]. AKI is an essential issue for patients scheduled to undergo cardiac surgery because those who develop AKI have an increased risk of renal replacement therapy (RRT), in-hospital mortality, and long-term mortality [4,5]. Carperitide, which is an atrial natriuretic peptide preparation, is expected to prevent AKI in cardiovascular surgery in Japan [6,7].

Carperitide has multiple independent mechanisms of action, including vasodilation, inhibition of sodium reabsorption, reduction of renin activity, angiotensin II concentrations, blood aldosterone concentrations, and sympathetic inhibition [8]. Continuous administration of carperitide exerts a potent natriuretic effect. Carperitide is also nephroprotective by increasing diuresis and the glomerular filtration rate in the prevention and treatment of perioperative AKI in cardiac surgery [8]. Many systematic reviews and meta-analyses with a high level of evidence have reported the benefits of carperitide [9]. However, the low quality of the included studies and the high heterogeneity of the results have not established carperitide as having a role in preventing and treating AKI [10-13]. Carperitide administration is not recommended in AKI practice guidelines [14], and what subset of patients carperitide is effective for is unclear. It has also been suggested that in the treatment of heart failure, the efficacy of carperitide may be stratified by the presence or absence of pulmonary congestion [15].

We hypothesized that in patients with pulmonary hypertension before cardiovascular surgery, the vasodilatory and diuretic effects of carperitide are beneficial, and the nephroprotective effects of carperitide are more effective than in those without pulmonary hypertension. Therefore, this study aimed to investigate whether there is a difference in the nephroprotective effect of carperitide during cardiac surgery between patients with preoperative pulmonary hypertension and those without pulmonary hypertension using our database.

## Materials and methods

We conducted a retrospective cohort study at Hamamatsu University Hospital (Shizuoka, Japan) between March 2019 and September 2021. The study protocol was approved (21-238) by the Ethics Review Board of Hamamatsu University School of Medicine. The Ethics Review Board waived the requirement for written informed consent because of the retrospective design of the study and the absence of follow-up. This study was conducted according to the STROBE checklist and complied with the Declaration of Helsinki 1964 and its later amendments.

We included adult patients aged 20 years or older who received carperitide (Hamp™; Daiichi-Sankyo, Tokyo, Japan) during cardiovascular surgery and were admitted to the intensive care unit (ICU) postoperatively. The exclusion criteria were as follows: (i) patients who did not have right ventricular catheterization or echocardiography before surgery; (ii) patients younger than 20 years of age; (iii) patients with emergency surgery; and (iv) patients who were not administered carperitide during surgery. According to the following procedure, all patients were classified into the pulmonary hypertension group and the control group without pulmonary hypertension. In this study, we used systolic pulmonary artery pressure as measured by preoperative right heart catheterization. In patients who did not undergo right heart catheterization, the estimated systolic pulmonary artery pressure was calculated from the tricuspid regurgitation pressure gradient and the estimated right atrial pressure on echocardiography [16]. Patients with a systolic pulmonary artery pressure of ≥36 mmHg were classified into the pulmonary hypertension group in accordance with the guideline [17]. In a sensitivity analysis, we changed the pulmonary hypertension group to those with a systolic pulmonary artery pressure of 50 mmHg using the guideline definition [17].

Demographic data (e.g., age, sex, body mass index, and Acute Physiology and Chronic Health Evaluation II score) were automatically extracted from our hospital data registered in the Japanese intensive care patient database (JIPAD) [18], which is a national ICU registry. Surgical information (e.g., type of surgery, duration of surgery, presence of a cardiopulmonary bypass, and carperitide dosage) was retrospectively examined manually by two independent authors in the anesthesia recording system ERGA (DOWELL, Sapporo, Japan). Information on outcomes (occurrence of AKI in the first 24 hours postoperatively, use of RRT in the ICU, urine volume in the first 24 hours postoperatively, and highest creatinine value in the first 24 hours postoperatively) was collected from JIPAD data. To maintain data accuracy and address potential bias, the data were collected by the first author (S.S.) and checked by the corresponding author (Y.A.).

The primary outcome was the incidence of AKI based on the Kidney Disease: Improving Global Outcomes (KDIGO) criteria within 24 hours after surgery [19]. Secondary outcomes were the number of patients using RRT in the ICU, urine volume within 24 hours after surgery, and the difference in serum creatinine concentrations between before and after surgery. Age, sex, body mass index, use of cardiopulmonary bypass, and the presence of blood transfusion were selected as confounders for multivariate analysis.

Statistical analyses

We calculated the median and interquartile range for continuous data and the number and percentage for categorical data. The Mann-Whitney test was applied to compare the mean values of continuous variables. Categorical variables were analyzed using the χ2 test or Fisher’s exact test. For the primary outcome, the incidence of AKI was analyzed using a multivariate logistic regression model. Sensitivity analysis was also based on multivariate logistic regression. The multivariate linear regression model was used to evaluate the secondary outcomes of continuous variables (urine volume within 24 hours after surgery and the difference in serum creatinine concentrations between before and after surgery). Additionally, the secondary outcomes of binary variables (number of patients using RRT) were evaluated using Fisher’s exact test because of the small number of events. Two-sided p values of <0.05 were considered statistically significant. All statistical analyses were conducted using Stata/BE 17.0 (Stata, College Station, TX, USA).

## Results

**Figure 1:**
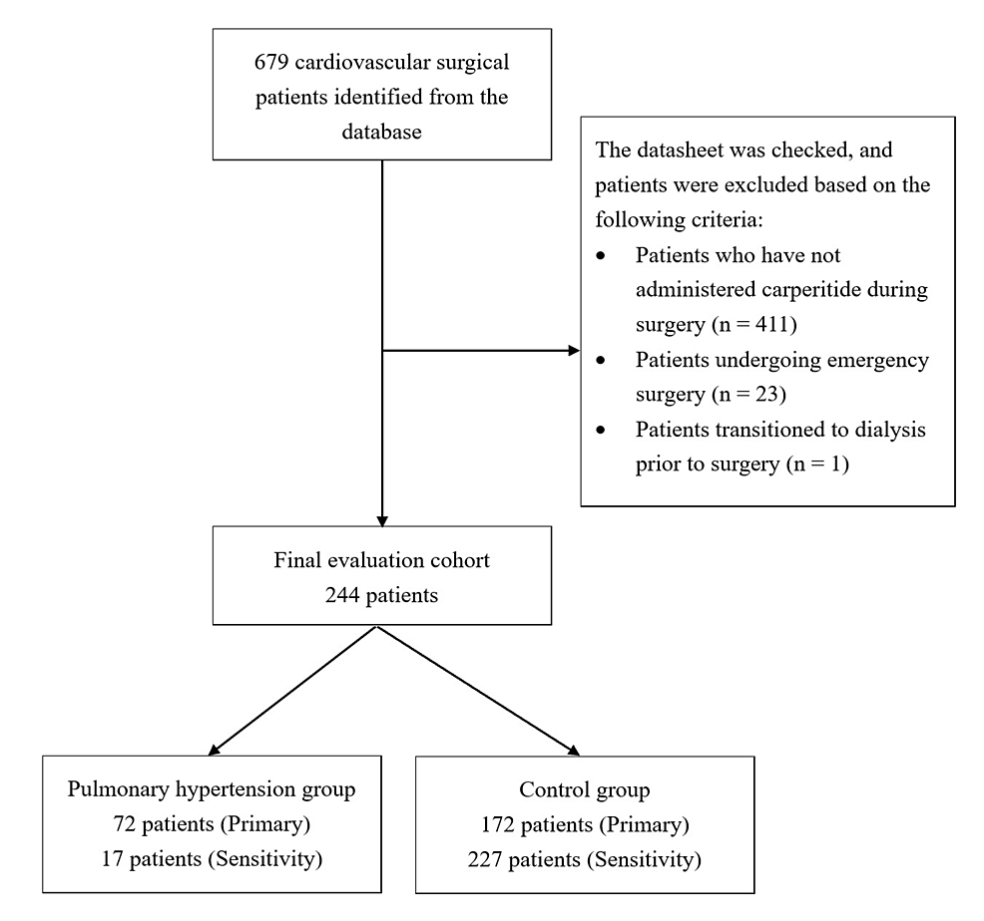
Flowchart of the study.

Six hundred seventy-nine patients were admitted to the ICU after cardiovascular surgery at Hamamatsu University Hospital during the study period. The exclusion criteria were applied (see Figure 1), and 244 patients were included in the final analysis. Seventy-six patients were investigated for pulmonary hypertension using right heart catheter systolic pulmonary artery blood pressure measurements, and 168 patients were investigated for pulmonary hypertension by calculating estimated systolic pulmonary artery pressure from echocardiography. In the primary analysis, an estimated systolic pulmonary artery pressure ≥36 mmHg was used as the cutoff, and the patients were classified into 72 patients in the pulmonary hypertension group and 172 patients in the control group. In a sensitivity analysis using estimated systolic pulmonary artery pressure ≥50 mmHg as the cutoff, 17 patients were in the pulmonary hypertension group, and 227 were in the control group.

The characteristics and surgical data of the patients included in this study are shown in Table 1. Patients were significantly older and had a lower rate of male sex in the pulmonary hypertension group than those in the control group (both p<0.05). Unsurprisingly, the estimated pulmonary artery pressure was significantly different between the two groups. The pulmonary hypertension group had a significantly lower infusion volume, less blood loss, and a lower transfusion volume than those in the control group (p=0.0017, p=0.0018, and p=0.024, respectively). The median dose of carperitide administered during surgery was 0.012 μg/kg/minute in both groups, with no difference between the groups.

**Table 1:** Characteristics and surgical data of patients who underwent cardiovascular surgery and were administered carperitide APACHE II, Acute Physiology and Chronic Health Evaluation II. Values are shown as number (%) or median (interquartile range).

	Pulmonary hypertension group (n=72)	Control group (n=172)	p
Age (years)	78 (72–84)	76 (68–80)	0.031
Male sex	40 (55.6%)	121 (70.4%)	0.026
Body mass index (kg/m^2^)	22.2 (19.7–24.7)	22.5 (20.0–25.4)	0.43
APACHE II score	17 (14–19)	15 (13–18)	0.062
Preoperative creatinine value (mg/dL)	1.10 (0.84–1.42)	1.01 (0.85–1.25)	0.42
Estimated pulmonary artery pressure (mmHg)	41 (39–48)	24 (14.5–30)	<0.001
Surgery time (minutes)	299 (120–425)	327 (246–423)	0.052
Anesthesia time (minutes)	390 (216–513)	428 (338–528)	0.052
Dose of carperitide (μg/kg/minute)	0.012 (0.0097–0.017)	0.012 (0.0094–0.017)	0.99
Infusion volume (mL)	1905 (1425–2500)	2300 (1625–3625)	0.0017
Urine volume (mL)	698.5 (390–1048)	877.5 (373–1469)	0.17
Blood loss (mL)	376 (20–887)	709.5 (226–1386)	0.0018
Transfusion volume (mL)	894.5 (0–1442)	1190.5 (560–1729)	0.024
Use of cardiopulmonary bypass	44 (61.1%)	118 (68.6%)	0.26
Cardiopulmonary bypass time (minutes)	214 (182–261)	191 (157–235)	0.059

**Figure 2:**
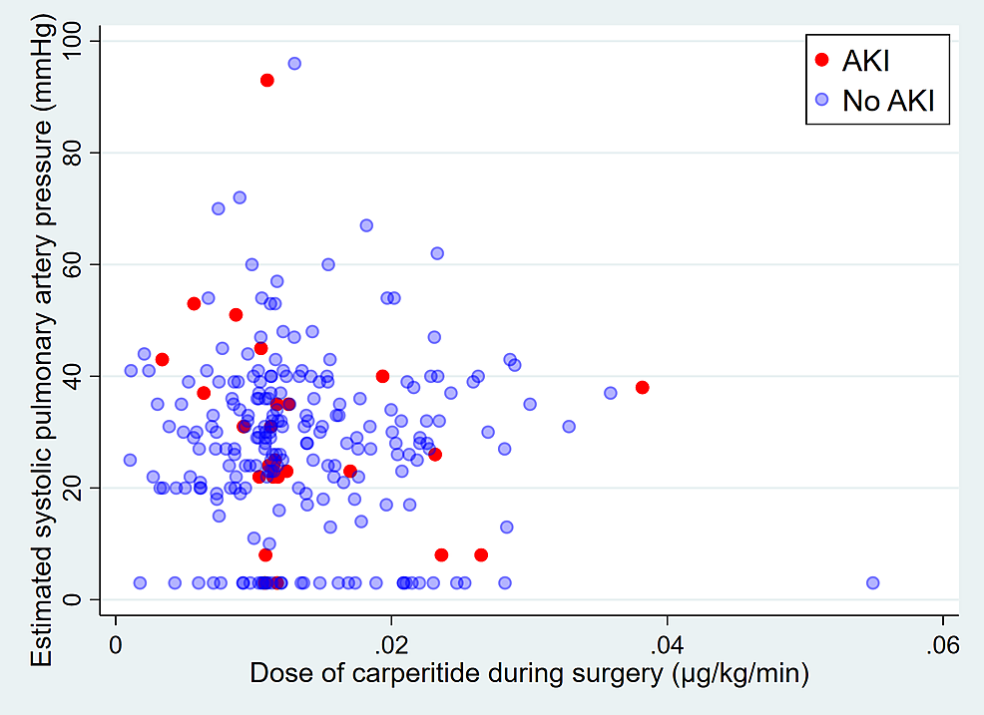
Relationship between the estimated systolic pulmonary artery pressure before surgery and the dose of carperitide administered during surgery. The median carperitide dose was <0.02 µg/kg/minute in the majority of patients. Red dots indicate patients who developed AKI; blue dots indicate patients who did not develop AKI. AKI, acute kidney injury.

Figure 2 shows the relationship between the estimated systolic pulmonary artery pressure before surgery and the dose of carperitide administered during surgery. When the patients were classified into the pulmonary hypertension and control groups using the estimated systolic pulmonary artery pressure of 36 mmHg as the cutoff, eight of 72 (11.1%) patients in the pulmonary hypertension group and 18 of 172 (10.5%) patients in the control group developed AKI (Table 2). Logistic regression analysis adjusted for confounding factors showed no significant difference in the incidence of AKI between the two groups (odds ratio 1.40, 95% confidence interval 0.54-3.62, p=0.49). A sensitivity analysis using an estimated systolic pulmonary artery pressure of 50 mmHg as the cutoff showed that three of 17 (17.7%) patients in the pulmonary hypertension group and 23 of 227 (10.1%) patients in the control group developed AKI. A logistic regression analysis adjusted for confounders showed no significant difference in the incidence of AKI between the two groups (odds ratio 1.96, 95% confidence interval 0.46-8.29, p=0.36).

**Table 2:** Clinical outcomes associated with kidney function AKI, acute kidney injury; RRT, renal replacement therapy. Values are shown as number (%) or median (interquartile range).

	Pulmonary hypertension group (n=72)	Control group (n=172)	p
AKI	8 (11.1%)	18 (10.5%)	0.49
Use of RRT	0 (0%)	3 (1.7%)	0.56
Urine volume within 24 hours (mL)	2458 (1589–3274)	2430 (1765–3186)	0.93
Creatinine difference (mg/dL)	-0.05 (-0.12–0.10)	0.03 (-0.08–0.12)	0.94
Highest creatinine value within 24 hours postoperatively (mg/dL)	1.10 (0.81–1.42)	1.06 (0.85–1.42)	0.92

The secondary outcomes are shown in Table 2. The number of patients using RRT was not significantly different by Fisher’s exact test between the two groups. The urine volume within 24 hours after surgery was not significantly different by multiple regression analysis between the groups (mean difference: −12.7 mL, 95% confidence interval −302 to 277). The difference in serum creatinine concentrations between before and after surgery was not significantly different by multiple regression analysis between the groups (mean difference: 0.0023, 95% confidence interval −0.061 to 0.066).

## Discussion

We aimed to investigate whether there is a difference in the nephroprotective effect of carperitide during cardiac surgery between patients with preoperative pulmonary hypertension and those without pulmonary hypertension. The incidence of AKI, which was the primary endpoint, was not different between the two groups and was robust in a sensitivity analysis. The number of patients using RRT in the ICU, urine volume within the first 24 hours after surgery, and the difference in serum creatinine concentrations were not different between the two groups. Therefore, carperitide had a similar effect according to the presence or absence of preoperative pulmonary hypertension in this study.

To the best of our knowledge, this study is the first to investigate whether there is a difference in the nephroprotective effect of carperitide according to the presence or absence of pulmonary hypertension. Although our hypothesis focusing on pulmonary hypertension was rejected, previous studies have reported preventing AKI by stratifying risk in the perioperative period of cardiac surgery [20,21]. Carperitide, which is an atrial natriuretic peptide preparation, has been widely administered and studied in Japan [22]. Carperitide has vasodilatory and natriuretic effects Therefore, low doses (0.01-0.05 µg/kg/minute) of carperitide are recommended because adverse effects of hypotension have been reported at standard doses (0.1-0.2 µg/kg/minute) [7,23,24]. The carperitide dose in our study was in the low dose category, but unfortunately, we could not stratify the nephroprotective effect of carperitide.

In this study, there were no differences in the number of patients using RRT, urine volume within 24 hours after surgery, or the difference in serum creatinine concentrations before and after surgery between patients with and those without pulmonary hypertension. Previous reports have suggested the efficacy of carperitide regarding the number of RRTs performed, urine volume, and serum creatinine values 24 hours after surgery [7,25-27]. We believe that the outcomes that we adopted to evaluate carperitide are reasonable.

The definition of pulmonary hypertension has not been standardized. We set the cutoff for pulmonary hypertension at 36 mmHg and also used 50 mmHg in a sensitivity analysis on the basis of the guideline [17]. However, the updated guidelines suggest a mean pulmonary artery pressure of 25 mmHg for the cutoff [28]. Additionally, we calculated the estimated pulmonary artery pressure according to echocardiography guidelines in patients without right heart catheters [16]. Estimating pressure in the right ventricular system is sometimes complex, and pulmonary artery pressure calculated from echocardiography may not be accurate in some situations [29,30]. Further research is required on the definition of pulmonary hypertension used in this study.

The present study has several limitations. The first limitation is that because this study was a single-center, retrospective study, there was no protocol for the method of carperitide administration. However, the carperitide dosage used at our institution was low, which is mostly consistent with previous reports [25-27]. Additionally, there were no differences in outcomes between the two groups. Second, because this study used data from the JIPAD, which is a national database on intensive care in Japan, the main results were available within 24 hours after surgery. The number of patients diagnosed with postoperative AKI may have been lower than estimated because elevated serum creatinine values can last up to seven days according to the KDIGO criteria [19]. However, we had the advantage of collecting data without loss in all cases, and for primary treatments such as RRT, we were able to collect data within the ICU stay. Third, either echocardiography or right heart catheterization made the diagnosis of pulmonary hypertension, and systolic pulmonary artery pressure was used to perform the primary and sensitivity analyses for both tests. In addition to challenges in the accuracy of the diagnosis of pulmonary hypertension, this study did not investigate the clinical presentation of the patients. Therefore, it is a limitation of retrospective studies and requires future prospective studies. Fourth, unknown confounding factors, such as administering other circulatory agonists and diuretics used intraoperatively and postoperatively, were not adjusted for. Additionally, the number of events, including the number of RRTs, was small, which may have prevented adequate adjustment for confounding factors. This issue may have been a problem in the study design and requires further investigation.

## Conclusions

We focused on the vasodilatory effect of carperitide and hypothesized that carperitide is more effective in patients with pulmonary hypertension than in those without pulmonary hypertension. Unfortunately, stratification by the presence or absence of pulmonary hypertension did not result in any differences in renal function outcomes, including the incidence of AKI, which is a measure of the nephroprotective effect of carperitide. However, the risk stratification of developing AKI has been a subject of recent investigation, and further research is warranted.
